# Shared functional neural substrates in Parkinson's disease and drug-induced parkinsonism: association with dopaminergic depletion

**DOI:** 10.1038/s41598-020-68514-0

**Published:** 2020-07-15

**Authors:** Se Won Oh, Na-Young Shin, Uicheul Yoon, Intae Sin, Seung-Koo Lee

**Affiliations:** 1 Department of Radiology, College of Medicine, The Catholic University of Korea, 222 Banpo-daero, Seocho-gu, Seoul, 06591 Korea; 2 Department of Radiology, Research Institute of Radiological Science, Yonsei University College of Medicine, 50-1 Yonsei-ro, Seodaemun-gu, Seoul, 120-752 Korea; 3 Department of Biomedical Engineering, College of Health and Medical Science, Catholic University of Daegu, Gyeongsan, Korea

**Keywords:** Parkinson's disease, Parkinson's disease, Parkinson's disease

## Abstract

While drug-induced parkinsonism (DIP) is mainly caused by blockage of the dopaminergic pathway, multiple neurotransmitter systems besides the dopaminergic system are involved in Parkinson’s disease (PD). Therefore, alterations found in both DIP and PD might be manifestations of dopaminergic dysfunction. To prove this hypothesis, we aimed to define the areas commonly involved in DIP and PD and determine whether the overlapping areas were associated with the dopaminergic system. 68 PD patients, 69 DIP patients and 70 age-and sex-matched controls underwent resting-state functional MRI (rsfMRI). Regional homogeneity (ReHo), amplitude of low-frequency fluctuation (ALFF) and fractional ALFF were calculated and compared. Afterwards, we compared mean rsfMRI values extracted from the overlapping areas with uptake quantitatively measured on dopamine transporter (DAT) images and neuropsychological test results. Compared to the controls, both PD and DIP patients revealed altered rsfMRI values in the right insular cortex, right temporo-occipital cortex, and cerebellum. Among them, decreased ALFF in the right insular cortex and decreased ReHo in the right occipital cortex were correlated with decreased DAT uptake in the caudate as well as executive, visuospatial, and language function. Increased ReHo in the cerebellum was also correlated with decrease DAT uptake in the posterior and ventral anterior putamen, but not with cognitive function. In conclusion, the insular cortex, occipital cortex, and cerebellum were commonly affected in both PD and DIP patients and might be associated with altered dopaminergic modulation.

## Introduction

Parkinson’s disease (PD) is the leading cause of parkinsonism which is a clinical syndrome characterized by resting tremors, rigidity, dyskinesia and postural instability^1–3^. The degeneration of dopaminergic neurons in the substantia nigra and dysfunction of the dopaminergic nigro-striatal pathway is the main pathophysiology of PD^4,5^. PD is also affected by changes in non-dopaminergic neural substrates such as serotonin, noradrenaline and acetylcholine, which contribute to its motor and non-motor symptoms^6^. Hitherto, many studies using resting-state functional magnetic resonance imaging (rsfMRI) have reported intrinsic functional changes in widespread brain regions in PD^7,8^, and these changes might be related to both dopaminergic and non-dopaminergic alterations.

Unlike PD, drug-induced parkinsonism (DIP), the second most common cause of parkinsonism, is generally caused by drugs blocking the dopamine D2 receptor in the striatum^9^. However, the effects of other mechanisms set off by a variety of offenders (e.g., presynaptic dopamine storage depletion or mitochondrial respiratory chain dysfunction)^10,11^ and various underlying diseases (i.e., psychotic disorder, depression, or epilepsy)^12^ can affect the intrinsic activation pattern of the brain together. Therefore, common features in the intrinsic brain activity pattern of PD and DIP patients might reflect dysfunctions in the dopaminergic system – especially in postsynaptic D2 receptor-related dopaminergic modulation—more specifically relevant to parkinsonian symptoms.

Defining dopamine-related regions relevant to parkinsonism is clinically valuable. Such knowledge can provide a basis for understanding its underlying pathophysiology, setting treatment targets, and monitoring treatment response.

So far, many researchers have tried to unveil the role of dopamine in the brain using positron emission tomography with dopamine receptor radioligands and task-based fMRI with or without combined drug challenges. However, few researchers have studied dopamine-related intrinsic functional alterations and most have focused on functional connectivity or network-level change^13–15^. Research on the regional influence of dopamine, which might be attributable to changes in functional connectivity or network-level change is still lacking.

Among several rsfMRI analytic methods, regional homogeneity (ReHo), amplitude of low-frequency fluctuation (ALFF), and fractional ALFF (fALFF) are data-driven methods and can be used to investigate the intrinsic activation patterns of each voxel^16^. ReHo measures how synchronously a particular voxel activates compared to its surrounding voxels^17^, and ALFF and fALFF represent the power of intrinsic brain activity in a particular voxel^18,19^.

So, we compared the intrinsic brain activity of PD and DIP patients to that of the controls, respectively, using the aforementioned rsfMRI methods. Then, we performed a conjunction analysis by extracting the overlapping areas in each comparison to define regions commonly involved in both patient groups. Additionally, to validate the association between functional change in the overlapping regions and dopamine depletion, we performed a correlation analysis between rsfMRI values in these areas and radioisotope uptake in the striatum on dopamine transporter (DAT) imaging, which represents presynaptic dopaminergic degeneration.

## Results

### Demographic characteristics

The demographic characteristics of the subjects are presented in Table 1. Years of education was significantly low in the DIP group compared to both the PD and control groups. The DIP group showed more symmetric motor symptoms, while the PD group showed more asymmetric involvement. In terms of the asymmetrically involved side, more severe symptoms were more frequently observed on the right side of PD patients, while being observed more frequently on the left side of DIP patients. The mean K-MMSE score was significantly different between groups and was highest for controls while being lowest for the DIP group. Both the PD and DIP groups showed decreased performance in executive, verbal and visual memory, visuospatial, and language functions. In addition, the DIP group showed decreased attention/working memory function compared to the control group. Compared to the PD group, the DIP group showed decreased performance in attention/working memory, visual memory, visuospatial, and language functions (Supplementary material Table S1).

**Table 1:** Demographic and clinical characteristics.

	PD (n = 68)	DIP (n = 69)	Control (n = 70)	*P* value	Post-hoc analysis		
					P1	P2	P3
Age (year)	69.7 ± 7.8	69.9 ± 7.7	67.8 ± 6.9	0.19			
Male	22 (32.4%)	15 (21.7%)	20 (28.6%)	0.628			
Education (year)	9.6 ± 4.6	6.6 ± 5.0	11.0 ± 5.6	< 0.001	0.002	0.235	< 0.001
UPDRS motor score	23.3 ± 9.5	24.5 ± 12.4		0.572			
Motor symptoms laterality				0.001			
Right > left	32	8					
Right < left	22	16					
Symmetric involvement	14	45					
Offending drugs							
Antiemetics		24 (34.8%)					
Antidepressants		12 (17.4%)					
Antipsychotics		4 (2.2%)					
Calcium channel blockers		10 (14.5%)					
Antiarrhythmics		1 (1.4%)					
Multiple		18 (26.1%)					
K-MMSE	26.6 ± 2.3	23.7 ± 4.8	28.5 ± 1.5	< 0.001	< 0.001	0.001	< 0.001

Unless otherwise indicated, data are presented as means ± standard deviations or numbers with percentages in parentheses. PD = Parkinson's disease; DIP = drug-induced parkinsonism; UPDRS = Unified PD Rating Scale; K-MMSE = Korean version of the mini-mental state examination. *P* values for comparison among the 3 groups, except for comparison of the UPDRS motor score and laterality for motor symptoms which were only compared between the PD and DIP groups. In the post-hoc analysis, *P*1 indicates *P* values for comparison between the PD and DIP groups, *P*2 for comparison between the PD and control groups and *P*3 for comparison between the DIP and control groups.

### Altered intrinsic brain activity in PD and DIP patients

#### ALFF

**Figure 1:**
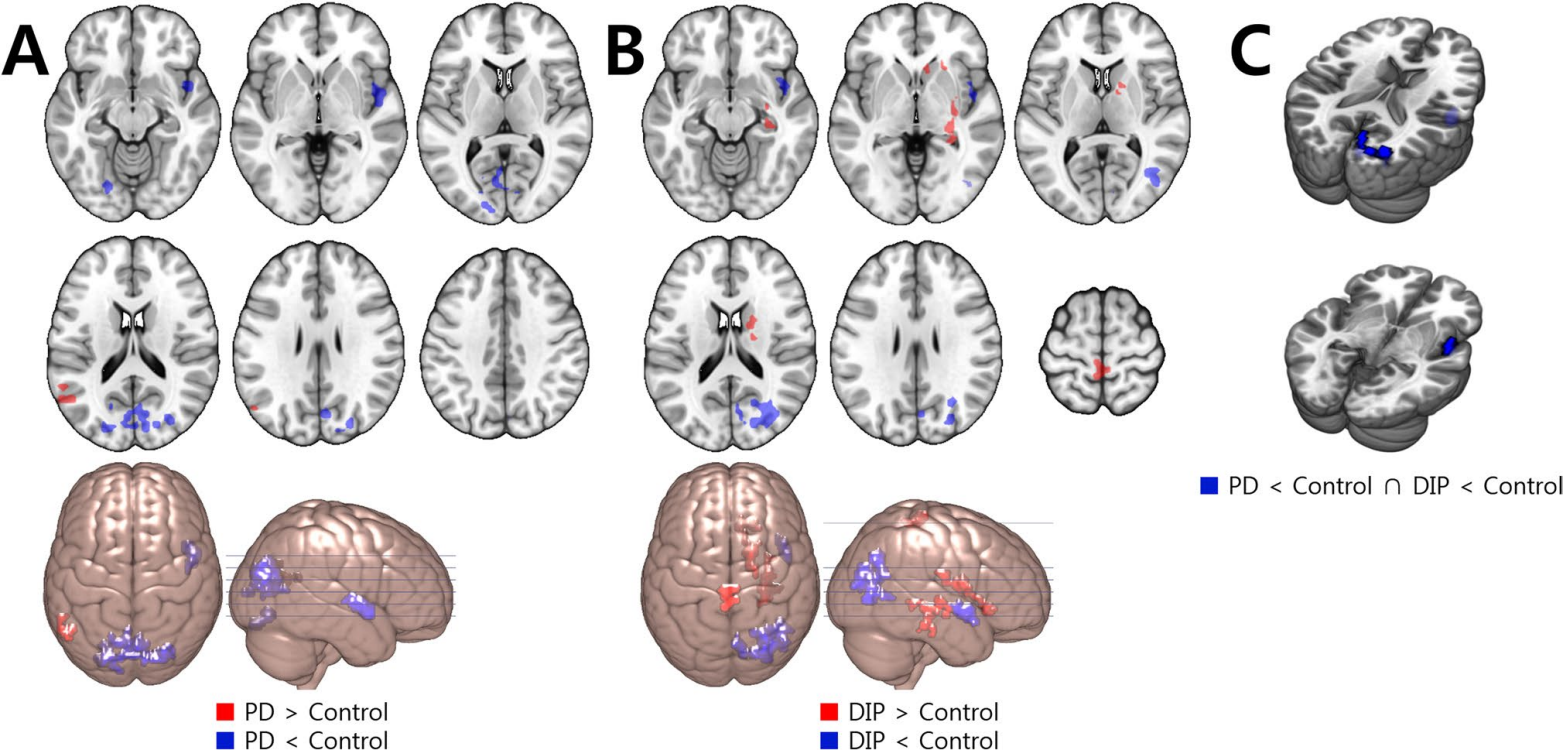
Regions with ALFF change compared to the control group. Red color indicates increased value and blue indicates decreased value. (**A**) The PD group showed decreased ALFF in the bilateral occipital area, right insular cortex and left cerebellum, and increased ALFF in the left parietal area. (**B**) The DIP group showed decreased ALFF in the right parieto-occipital area and right insular cortex and increased ALFF in the bilateral paracentral lobule, right striatum and right temporal area. (**C**) The right occipital and insular cortex showed decreased ALFF in both patient groups.

Compared to the control group, the PD group showed decreased ALFF in the bilateral occipital area, right insular cortex and left cerebellum, and increased ALFF in the left parietal area. The DIP group showed decreased ALFF in the right parieto-occipital area and right insular cortex and increased ALFF in the bilateral paracentral lobule, right striatum and right temporal area, compared to the control group (Fig. 1 and Supplementary material Table S2). Therefore, both the PD and DIP groups showed decreased ALFF in the right insular cortex and right occipital area compared to the control group (Fig. 1 and Table 2).

**Table 2:** Talairach coordinates and number of voxels for overlapping regions which show common changes of each rsfMRI value for the PD and DIP groups compared to the controls.

Analysis	Contrast	Region	Number of voxels	Talairach coordinates		
				**X**	**Y**	**Z**
ALFF	DIP < control and PD < control	R cuneus/middle occipital cortex	71	10.0	− 79.0	11.0
	DIP < control and PD < control	R insula/superior temporal gyrus	31	43.0	− 1.0	− 6.0
fALFF	DIP < control and PD < control	R mid temporal gyrus	19	41.0	− 69.0	4.0
ReHo	DIP < control and PD < control	R cuneus	3	10.0	− 64.0	16.0
		R cuneus	2	12.0	− 58.0	14.0
	DIP > control and PD > control	R cerebellum	33	4.0	− 54.0	− 43.0

*R *right.

#### fALFF

**Figure 2:**
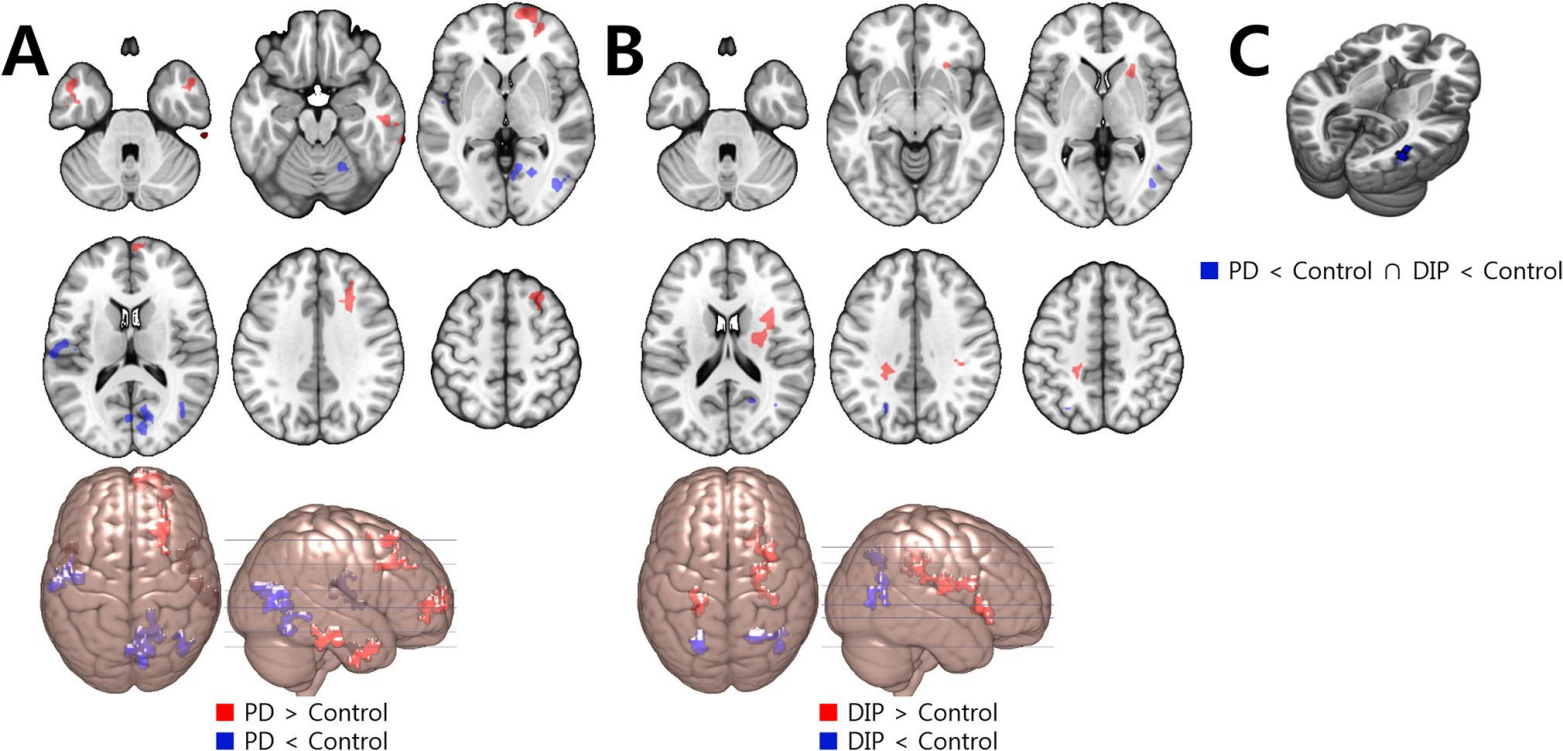
Regions with fALFF change compared to the control group. Red color indicates increased value and blue indicates decreased value. (**A**) The PD group showed decreased fALFF in the left inferior frontal and right temporo-occipital area, and increased fALFF in the right frontal and bilateral temporal area. (**B**) The DIP group showed decreased fALFF in the bilateral parietal and right temporal area and increased fALFF in the right striatum and bilateral corona radiata. (**C**) The right temporal cortex showed decreased fALFF in both patient groups.

The PD group showed decreased fALFF in the left inferior frontal and right temporo-occipital area and increased fALFF in the right frontal and bilateral temporal area, compared to the control group. The DIP group showed decreased fALFF in the bilateral parietal and right temporal area and increased fALFF in the right striatum and bilateral corona radiata (Fig. 2 and Supplementary material Table S3). So, fALFF in the right temporal area was significantly decreased in both patient groups (Fig. 2 and Table 2).

#### ReHo

**Figure 3:**
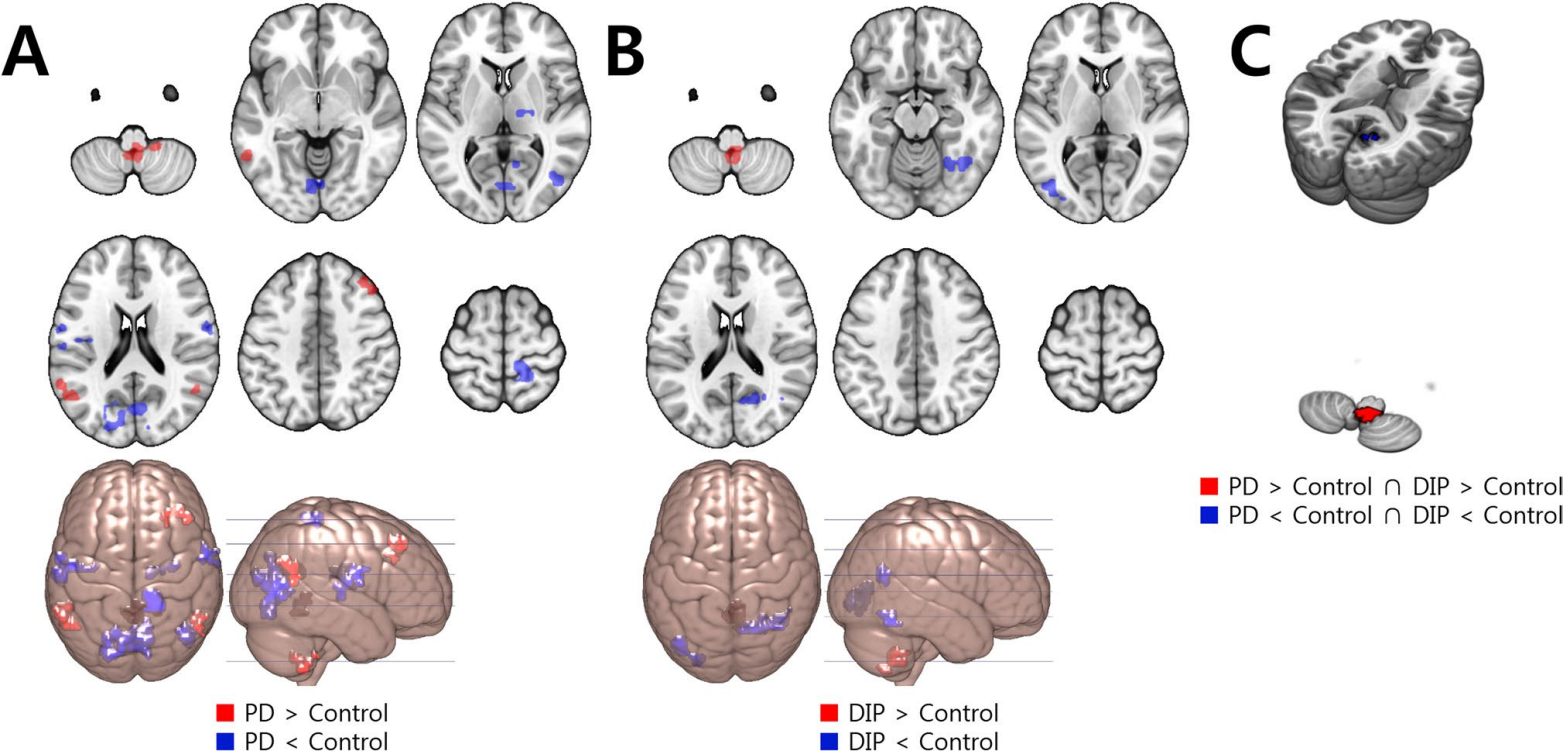
Regions with ReHo change compared to the control group. Red color indicates increased value and blue indicates decreased value. (**A**) The PD group showed decreased ReHo in the bilateral occipital cortex, and increased ReHo in the bilateral temporal and right frontal cortices and bilateral cerebellum. (**B**) The DIP group showed decreased ReHo in the bilateral temporo-occipital cortex and increased ReHo in the bilateral cerebellum. (**C**) The right occipital cortex showed decreased ReHo in both groups and the bilateral cerebellum showed increased activity in both groups.

Compared to the control group, the ReHo of the PD group was decreased in the bilateral occipital cortex and increased in the bilateral temporal and right frontal cortices and bilateral cerebellum. The DIP group showed decreased ReHo in the bilateral temporo-occipital cortex and increased ReHo in the bilateral cerebellum (Fig. 3 and Supplementary material Table S4). Therefore, the ReHo was decreased in the right occipital cortex and increased in the bilateral cerebellum in both patient groups relative to the control group (Fig. 3 and Table 2).

### Association between altered intrinsic brain activity and nigrostriatal dopaminergic depletion in PD patients

There was no significant difference for rsfMRI values between the 59 PD patients included in the correlation analysis and the 9 PD patients excluded from the analysis. In the included 59 PD patients, all five striatal subregions showed significant decrease in DAT uptake compared to the DIP patients (Supplementary material Table S5). Decreased DAT uptake in the caudate subregion was associated with decreased ALFF in the right insular cortex and decreased ReHo in the right occipital cortex. Decreased DAT uptake in the posterior and ventral anterior putamen was associated with increased ReHo in the bilateral cerebellum (Table 3).

**Table 3:** Correlation analysis between rsfMRI values in areas showing common change and striatal DAT uptake.

	fALFF in right temporal ROI	ReHo in right occipital ROI	ReHo in cerebellar ROI	ALFF in right insular ROI	ALFF in occipital ROI
Ventral caudate	0.248 (0.058)	.300 (0.021)*	− 0.199 (0.13)	.302 (0.02)*	0.092 (0.489)
Dorsal caudate	0.24 (0.067)	.332 (0.01)*	− 0.189 (0.152)	.327 (0.012)*	0.051 (0.701)
Posterior putamen	− 0.006 (0.966)	0.056 (0.675)	− .330 (0.011)*	0.058 (0.664)	0.034 (0.796)
Ventral anterior putamen	0.114 (0.389)	0.177 (0.181)	− .301 (0.021)*	0.198 (0.133)	0.132 (0.318)
Dorsal anterior putamen	0.025 (0.853)	0.171 (0.195)	− 0.223 (0.089)	0.208 (0.114)	0.158 (0.233)

Data are presented as the Pearson’s *r* with *P* values in parentheses.

*P < .05.

### Association between altered intrinsic brain activity and cognitive function

There was no significant difference for rsfMRI values between the 90 subjects included in this correlation analysis and the 47 subjects excluded from it. In the 90 included subjects, decreased ALFF in the right insular cortex was associated with poor performance in the COWAT_supermarket (*r* = 0.257, *P* = 0.015). Decreased ReHo in the right occipital cortex was associated with poor performance in the COWAT_supermarket (*r* = 0.247, *P* = 0.019), RCFT copying (*r* = 0.277, *P* = 0.008), and K-BNT (*r* = 0.214, *P* = 0.042). Decreased fALFF in the right temporal area was associated with better performance in the RCFT immediate recall test (*r* = − 0.223, *P* = 0.035). Complete results of correlation analysis are presented in Table 4.

**Table 4:** Correlation analysis between functional activity in areas showing common change and neuropsychological data.

Variables	ALFF in right temporal ROI	ReHo in right occipital ROI	ReHo in cerebellar ROI	ALFF in right insular ROI	ALFF in occipital ROI
Digit span (forward)	− 0.195 (0.065)	0.187 (0.077)	0.093 (0.384)	0.044 (0.678)	− 0.219 (0.038)
Digit span (backward)	− 0.046 (0.664)	0.308 (0.003)**	0.001 (0.991)	0.13 (0.221)	− 0.067 (0.53)
Digit span total	− 0.137 (0.197)	0.275 (0.009)**	0.054 (0.614)	0.096 (0.366)	− 0.162 (0.126)
Word Stroop test	− 0.104 (0.339)	0.087 (0.428)	− 0.068 (0.532)	0.001 (0.992)	− 0.064 (0.56)
Color Stroop test	0.201 (0.064)	0.394 (0)**	− 0.076 (0.488)	0.306 (0.004)**	− 0.083 (0.45)
Phonemic generative naming	0.043 (0.701)	0.184 (0.098)	0.051 (0.648)	0.022 (0.847)	− 0.01 (0.932)
COWAT (animal)	− 0.083 (0.439)	0.185 (0.082)	− 0.075 (0.482)	0.103 (0.333)	0.179 (0.092)
COWAT (supermarket)	− 0.02 (0.852)	0.247 (0.019)*	− 0.073 (0.495)	0.257 (0.015)*	0.137 (0.196)
SVLT free recall	− 0.019 (0.857)	0.168 (0.113)	− 0.023 (0.828)	0.083 (0.439)	0.149 (0.16)
SVLT delayed recall	− 0.157 (0.14)	0.073 (0.493)	− 0.134 (0.209)	0.085 (0.427)	0.038 (0.726)
SVLT recognition	0.016 (0.884)	− 0.03 (0.777)	− 0.175 (0.099)	− 0.017 (0.875)	0.074 (0.487)
RCFT immediate recall	− 0.223 (0.035)*	0.119 (0.262)	− 0.053 (0.619)	0.003 (0.975)	− 0.058 (0.587)
RCFT delayed recall	− 0.162 (0.128)	0.13 (0.222)	− 0.068 (0.526)	0.017 (0.872)	− 0.091 (0.392)
RCFT recognition	− 0.114 (0.283)	− 0.122 (0.25)	− 0.025 (0.815)	− 0.078 (0.463)	0.047 (0.661)
RCFT copy	− 0.164 (0.124)	0.277 (0.008)**	0.001 (0.996)	0.049 (0.648)	− 0.085 (0.427)
K-BNT	− 0.093 (0.382)	0.214 (0.042)*	− 0.115 (0.282)	0.019 (0.856)	− 0.044 (0.681)

Data are presented as the Pearson’s *r* with the *P* values in parentheses.

*SVLT* Seoul Verbal Learning Test, *COWAT* Controlled Oral Word Association Test, *RCFT* Rey Complex Figure Test, *K-BNT* Korean version of the Boston Naming Test.

*P < .05; **P < .01.

## Discussion

In the present study, we noted changes in intrinsic functional activity in the right insular cortex, right temporo-occipital cortex and cerebellum, common to both groups. Decreased rsfMRI values in the right insular and right occipital cortices were correlated with decreased DAT uptake in the caudate as well as executive, visuospatial, and language functions. Increased rsfMRI values of the cerebellum were also correlated with decrease DAT uptake in the posterior and ventral anterior putamen, but not with cognitive function. Therefore, the insular cortex, occipital cortex, and cerebellum might be associated with altered dopaminergic modulation in both PD and DIP patients.

Accumulating evidence suggests that the insula—specifically the anterior part—plays a cognitive hub in high-level cognitive control and directing cognitive processes by switching between large-scale brain networks relevant to executive function^20,21^. This function of the insula can be disrupted by dopaminergic dysfunction via the D2 receptor in the insula (e.g., blocking the D2 receptor with drugs in DIP)^12,22^ or loss of dopaminergic modulation in the basal ganglia which is anatomically highly interconnected with the insula (e.g., presynaptic degeneration of the nigrostriatal dopaminergic neuron in PD)^23^. As one might expect, previous research showed a relationship between striatal dopaminergic depletion, decreased D2 receptor availability in the right insula, and decreased executive function in PD patients^24^. Our results also showed decreased ALFF in the right insula in both patient groups. Moreover, decreased ALFF in the right insula was correlated with dopaminergic depletion of the caudate and decreased executive function, supporting earlier results showing the insula being modulated by dopamine.

Decreased ReHo in the right occipital cortex, near the visual cortex, was also correlated with dopaminergic depletion in the caudate. Cortical thinning^25^, decreased metabolism^26,27^, and decreased connectivity^28^ in the occipital cortex have been reported even in early PD, reflecting visual dysfunction found in this population^29^. Autopsy studies have found dopaminergic innervation in the visual cortex of humans without neurological disease^30^ and decreased dopamine concentration in the retina, which is connected to the occipital cortex via the visual pathway, in PD patients^31^, which suggests that dopaminergic modulation might play a role in changes in the occipital cortex. However, the underlying pathophysiology for these changes are still not completely understood and further research is warranted.

Although the role of the cerebellum in PD also remains unclear, hyperactivity^32^, hyperconnectivity^33^, hypermetabolism^34^, and volume loss of the cerebellum^35,36^, which have been associated with motor and non-motor symptoms, have been repeatedly reported in recent studies, suggesting that the cerebellum plays a substantial role in clinical manifestations of PD. Fewer studies have been performed on the role of the cerebellum in DIP than PD, and a previous study^3^ only reported hypertrophy of the cerebellum in DIP compared with controls. As the cerebellum also has dopaminergic receptors (D1–3)^37,38^ and is anatomically connected with the substantia nigra via dopaminergic projection^39^, dopamine might be able to modulate cerebellar function and structure. Our study also revealed increased ReHo in the cerebellum including the uvula of vermis, for which a previous study found decreased levels of dopamine D1 and D3 messenger RNA in PD^37^, and the ReHo in the cerebellum was negatively correlated with dopaminergic depletion in the putamen. These functional, metabolic, and structural changes might be a compensatory process of the cerebello-thalamo-cortical loop for the hypofunction of the striato-thalamo-cortical circuit^40,41^. These changes could also be pathological changes induced by dopaminergic degeneration or abnormal stimulation from a subthalamic nucleus disinhibited by striatal D2 receptor blockage or presynaptic dopaminergic degeneration. Unlike previous studies, we could not find significant associations between cerebellar change and cognitive and motor function (not shown). The mixed pathophysiology underlying cerebellar change might be a possible explanation, but further investigation is needed to elucidate the clinical impact of cerebellar change. We also noted decreased amplitude of intrinsic activity in the posterior temporo-occipital cortices in both PD and DIP, but without correlation with dopaminergic depletion. The functional change in the posterior brain cortex has been suggested to be mainly affected by the cholinergic system, rather than the dopaminergic system^42^. As seen in previous articles^43–45^, DIP patients may be in a preclinical stage of PD and their parkinsonism may have been unmasked by offending drugs. Therefore, unmasked preclinical PD manifestation with a disturbed cholinergic system in DIP patients might be a possible explanation for the decreased ALFF and fALFF values observed in the posterior cortical areas in our study.

The basal ganglia plays a key role in the pathogenesis of both PD and DIP^4,5,9^. But in our study, there were no overlapping functional changes observed in the basal ganglia of both groups. In a previous longitudinal study with a follow-up of approximately 2 years, the basal ganglia did not show functional changes in the early stages of PD, but showed decreased activity in the later stages^46^. The PD patients enrolled in our study were drug-naïve and in the early stages of PD. We think this might have at least partly affected our results, although the size of this effect cannot be estimated at this time.

Another noteworthy finding is that the overlapping functional changes were quite asymmetric, involving mainly the right hemisphere. In terms of the laterality of motor symptoms, PD and DIP patients showed different patterns, so, it is difficult to explain the asymmetric results of our study simply with symptom laterality. Although asymmetric structural and functional brain changes have also been reported in previous imaging studies on PD^47–49^, the underlying mechanism remains unclear. Further study is necessary to elucidate the underlying pathophysiology of asymmetric brain involvement.

There are several limitations in this study. First, many drugs that induce DIP are antipsychotics. We didn’t consider the underlying psychiatric problems that require the use of antipsychotics. So, in some cases, the underlying psychiatric disease could have affected the resting-state activity of the brain. Also, not all patients were included in the correlation analyses between intrinsic brain activity and DAT uptake and between activity and NP test results, so this could cause a selection bias. However, we compared values of intrinsic brain activity between the included and excluded subjects, and found no significant difference.

In conclusion, we demonstrated common changes of intrinsic activity in PD and DIP patients using rsfMRI analysis. Both groups showed altered activities in the insular cortex, medial occipital cortex and cerebellum and these functional changes were correlated dopaminergic depletion, suggesting these areas as regions relevant to parkinsonism affected via dopaminergic modulation.

## Methods

### Subjects

Between August 2011 and June 2016, age-and sex-matched 68 drug-naïve PD patients, 69 DIP patients and 70 controls who underwent MRI were recruited. PD was diagnosed according to the clinical criteria of the United Kingdom (UK) PD Society Brain Bank^50^. DIP was diagnosed with the following criteria: (1) presence of at least two of the four cardinal signs of PD (i.e., resting tremor, rigidity, dyskinesia and postural instability), (2) absence of a history of extrapyramidal disorders prior to treatment with an offending drug, (3) onset of parkinsonian symptoms during treatment with the offending drug, and (4) symptom resolution or significant improvement after ceasing the offending drug. To ensure clinical diagnostic accuracy, only patients who revealed decreased uptake in the posterior putamen on DAT imaging were included in the PD group, while those with normal uptake were included in the DIP group. Patients with focal brain lesions, multiple old lacunes in the basal ganglia, or extensive white matter hyperintensities out of the normal range were excluded. Patients with Parkinsonian plus syndromes or other neurodegenerative diseases and medical comorbidities that might contribute to cognitive dysfunction were also excluded. Participants who had no active neurologic or psychologic disorders and no cognitive complaints with a minimal score of 27 on the Korean version of the mini-mental state examination (K-MMSE) were included in the control group. All data were collected in accordance with relevant guidelines and regulations.

### Standard protocol approvals, registrations, and patient consents

The Institutional Review Board of Severance Hospital approved this retrospective study and waived the need for informed consent as part of approval since we used retrospective de-identified data collected during outpatient visits. In addition, all methods were performed in accordance with the approved guidelines.

### Assessment of clinical information

The Unified PD Rating Scale Part III (UPDRS-III) was used to assess parkinsonian motor symptoms in all PD patients and 46 of the DIP patients. We separately summed up the scores for resting tremor, active tremor, rigidity, finger tapping, hand movement, and rapid alternative movements of hand and leg agility for each side according to UPDRS III. Then we defined functional changes as asymmetric when the difference between the right and left scores was more than two points, as was done in a previous study^51^. The self-rating Beck Depression Inventory^52^ was used to assess depressive symptoms in patients with PD (n = 66) and DIP (n = 49). A history of vascular risk factors including hypertension, diabetes mellitus, hyperlipidemia, and smoking was also investigated.

To assess cognitive status, the Seoul Neuropsychological Screening Battery (SNSB)^53^, a detailed neuropsychological (NP) test, was used in 66 of the PD patients, 33 of the DIP patients and 45 of the controls. The SNSB is comprised of (1) the forward and backward digit span tests and Stroop Test (word and color reading of 112 items during a 2-min period) for attention/working memory, (2) phonemic and semantic Controlled Oral Word Association Test (COWAT) for frontal/executive function, (3) Seoul Verbal Learning Test (SVLT; immediate recall, 20-min delayed recall, and recognition) and Rey Complex Figure Test (RCFT; immediate and 20-min delayed recall, and recognition) for verbal/visual memory, (4) the Korean version of the Boston Naming Test (K-BNT) for language, and (5) RCFT copying for visuospatial function.

### Image acquisition

All participants underwent fMRI scanning with a 3.0T MRI scanner (Achieva, Philips Medical System, Best, Netherlands) to obtain T2*-weighted single-shot echo-planar imaging sequences with the following parameters: 165 axial volume scans: voxel size, 2.75 × 2.75 × 3.0 mm^3^; slice number, 31 (interleaved); matrix, 80 × 80; slice thickness, 3.0 mm; gap, 1.0 mm; repetition time (TR), 2000 ms; echo time (TE), 30 ms; and field of view (FOV), 220 mm. Subjects were instructed to rest and keep their eyes closed without sleeping, moving, or thinking about anything specific for 5 min 30 s.

A 3D-T1-turbo field echo sequence was also obtained with the following parameters: axial acquisition with FOV, 220 mm; voxel size, 0.98 × 0.98 × 1.2 mm^3^; TR, 9.6 ms; TE, 4.6 ms; flip angle, 8°; no gap; and total acquisition time, 5 min 29.3 s.

DAT scans were performed with a GE PET-CT DSTe scanner (GE Discovery STE, GE Healthcare Technologies, Milwaukee, WI), which obtained images with a 3D-resolution of 2.3 mm thickness at half-maximum. All participants fasted for at least 6 h before DAT scanning. Each patient received 5 mCi (185 MBq) of 18F-FP-CIT intravenous injection, and images were acquired in the 3D mode at 120 KVp and 380 mAs during a 20-min session that took place 90 min after each injection.

### Image analysis

#### rsfMRI imaging preprocessing

As was done in our recently published study^16^, rsfMRI preprocessing and statistical analyses were performed using AFNI (https://afni.nimh.nih.gov/), MATLAB-based statistical parametric mapping software (SPM8, Wellcome Department of Imaging Neuro-Science, London, U.K.) and Data Processing Assistant for Resting-state fMRI (DPARSF, version 4.1; https://www.restfmri.net). We first despiked EPI data with AFNI’s 3d Despike algorithm to reduce the impact of outliers. The first 3 volumes of each image were discarded for magnetization stabilization, and the remaining 162 images were included in further analyses. Slice-timing correction was done by resampling all slices relative to the middle slice (i.e., 31st slice) in temporal order. When all functional volumes were realigned to the first volume to correct for head movement in SPM, six head motion parameters (three for translation and three for rotation) were calculated by interpolating movement over time during acquisition^54^. A maximal displacement of more than 3 mm or 3 degrees during an entire imaging scan was exclusion criteria for this study, but no data exceeded the criteria. Next, the functional images were co-registered to the T1-weighted image and spatially normalized to the Montreal Neurological Institute (MNI) template provided with SPM8, then resampled into 3-mm cubes. Smoothing was then performed only for ALFF and fALFF analyses with a 4-mm full-width, half-maximum (FWHM) isotropic Gaussian kernel. Functional images for ReHo analysis were not smoothed, but subsequent processes were identical to those of ALFF and fALFF analyses. Band-pass filtering (0.01–0.08 Hz) was then applied to the time series to reduce possible high frequency noise and signal drift, and each voxel was detrended to remove linear trends. Linear regression was estimated with six motion parameters, the CSF signal, the white matter (WM) signal, and the global mean as covariates to remove the head motion effect and other possible artifacts.

#### rsfMRI data analysis

Each rsfMRI value was calculated with the same methods used in our previous study^16^. ReHo represents the degree of regional similarity in intrinsic brain activation and is assessed by calculating Kendall’s coefficient of concordance (KCC) for the time series of a given voxel with those of its nearest 26 neighbors in a voxel-wise manner^55^. The formula for calculating the KCC is as follows:

$$ReHo = \frac{{\sum \left( {R_{i} } \right)^{2} - {\text{n}}\left( {\overline{R}} \right)^{2} }}{{K^{2} \left( {n^{3} - n} \right)/12}}$$

*K* is the number of voxels within each cluster, *n* is the number of ranks, and *Ri* is the sum of the ranks at the *i*th time point^17^. The KCC value can range from 0 to 1, and values closer to 1 indicate high similarity within a calculated cluster.

ALFF and fALLF represent the power or amplitude of intrinsic brain activity in a given voxel. To calculate ALFF, we transformed the time series for each voxel into the frequency domain using a fast Fourier transform after band-pass filtering and then obtained the power spectrum. As the square of the amplitude of a given frequency component is proportional to the power of this frequency, the square root was calculated at each frequency of the power spectrum and the averaged square root was then calculated across 0.01–0.08 Hz. This averaged square root was defined as ALLF. fALFF, the normalized index of ALFF, is defined as the ratio of a power within a specific low-frequency range to that of the entire detectable frequency range, and is considered to better reflect the resting-state of the brain than ALFF^56^. To reduce the global effects of variability across participants, each result map was respectively normalized by the global mean of the ReHo, ALFF and fALFF values.

To define differences in ReHo, ALFF, and fALFF between PD and DIP patients compared to the control group, respectively, we conducted pair-wise *t*-tests between the three groups in a voxel-wise manner using ANOVA contrast including age, sex, duration of education, and MMSE scores as covariates of no interest. All statistical analyses were corrected for multiple comparisons based on Monte Carlo simulations (3dClustSim, AFNI, https://afni.nimh.nih.gov). The results were set at an individual *P* value < 0.005 and a cluster size of 40 voxels corresponding to a corrected *P* < 0.05/3 in order to correct for the total number of comparisons between the three groups by applying a Bonferroni-like adjustment to the *t*-test significance level. Afterwards, a conjunction analysis was performed by extracting intersection in the comparative analysis results (PD vs. control and DIP vs. control) to identify areas showing altered activation common to PD and DIP patients compared to the control group.

### Quantitative analysis of DAT imaging data

The following image processing steps were applied based on a previous study in our institution^57^ to define a ROI from the DAT imaging data automatically, as described in detail elsewhere^58–62^. At first, the native MR images of all subjects were registered onto a template using a linear transformation and corrected for intensity non-uniformity artifacts^58^. All non-brain parts of the image including the skull and meninges were removed by an automated brain extraction algorithm^59^. A hierarchical multi-scale non-linear fitting algorithm was then applied (1) to normalize the individual MR images in stereotaxic space, (2) to provide a priori information, i.e. tissue probability maps for subsequent tissue classification using a neural network classifier, and (3) to obtain the 3D deformation vector field that maps the individual brain volume onto the template^61,62^. An artificial neural network classifier was applied to identify gray matter (GM), WM and CSF. Partial volume errors (PVE) due to tissue-mixing at their interfaces were estimated and corrected using trimmed minimum covariance determinant method^60,61^. A maximum probability atlas defining all of the major lobes and subcortical ROIs of the brain was warped to match each subject, and the intersection of this atlas with GM and WM classifications yielded striatal ROI masks including the caudate and putamen which were used to analyze the DAT imaging data^63^. We then divided the caudate nucleus into ventral (z ≤ 0) and dorsal (z > 0) parts, the putamen into anterior (y > 0) and posterior (y ≤ 0) parts, and the anterior putamen into ventral (z ≤ − 4) and dorsal (z > 0) parts based on the MNI space coordinates^64,65^ (Supplementary material Fig. S1). A GM mask was generated and included as an explicit mask to perform group comparisons exclusively within the GM voxels.

Individual DAT images were co-registered to the corresponding MRI using a rigid body transformation in order to apply a subcortical ROI mask. All voxels in the co-registered DAT images were divided by the mean uptake of the occipital GM over an established threshold (0.9) of GM PVE in each subject to form the specific to nonspecific binding ratio (SNBR) image. Finally, a regional mean SNBR was calculated in each striatal ROI.

### Correlation analysis between rsfMRI values and DAT uptake in PD patients

To define whether altered functional activities common to both PD and DIP patients compared to the control group were associated with dopaminergic depletion, we performed a correlation analysis between the rsfMRI values in the overlapping ROIs and SNBR in the striatal ROIs. We included only 59 PD patients who underwent DAT scans and MR scanning at an interval of 2 months or less in this analysis as DIP patients have normal DAT uptake in the striatum and as such, correlation results would not adequately reflect dopaminergic depletion in the brains of these patients.

### Correlation analysis between rsfMRI values and NP test results

Correlation analyses were performed to assess the relationship between the rsfMRI values in the overlapping ROIs and NP test results which showed remarkable differences in both the PD and DIP groups compared to the control group. To reduce the possibility of change in cognitive status on the MR scanning date compared to that on the NP test date, patients (61 PD and 29 DIP) with an interval of 2 months or less between the rsfMRI and NP tests were included for analysis. For each ROI, mean values were extracted from the ALFF, fALFF, and ReHo z-score maps with a 4-mm radius sphere centered at the peak. Then, correlation coefficients between the z values and the NP tests results were calculated. Two-tailed *P* values < 0.05 were considered significant.

### Statistical analysis

To compare the demographic characteristics of study subjects between the three groups, the χ^2^ test and Kruskal–Wallis test were used for categorical and continuous variables, respectively. For two-group comparisons, the independent two-sample *t*-test and Mann–Whitney *U* test were used according to the normality status confirmed by the Kolmogorov–Smirnov test. All data in the comparison analyses are expressed as means (standard deviations [SDs]). All correlation analyses were examined by Pearson’s correlation coefficient. Statistical analyses were performed using commercially available software (SPSS, Ver.24.0), and a two-tailed *P* value < 0.05 was considered significant.

The datasets generated during and/or analyzed during the current study are available from the corresponding author upon request.

## Supplementary information

Supplementary file1.
