# Supplementary material for: Shared functional neural substrates in Parkinson's disease and drug-induced parkinsonism: association with dopaminergic depletion
Source: Sci Rep. 2020 Jul 15;10:11617. doi: 10.1038/s41598-020-68514-0 (PMC7363811; doi:10.1038/s41598-020-68514-0)
Supplement: Supplementary file 1 — Supplementary file1. [file 41598_2020_68514_MOESM1_ESM.docx]

**Title**: Shared functional neural substrates in Parkinson's disease and drug-induced parkinsonism: association with dopaminergic depletion

**Authors**

Se Won Oh^1,2^, Na-Young Shin^1,2^, Uicheul Yoon^3^, Intae Sin^3^, Seung-Koo Lee^2^

**Author's institutional affiliations**

^1^Department of Radiology, College of Medicine, The Catholic University of Korea

^2^Department of Radiology, Research Institute of Radiological Science, Yonsei University College of Medicine

^3^Department of Biomedical Engineering, College of Health and Medical Science, Catholic University of Daegu

**Corresponding Authors**

Na-Young Shin M.D. Ph.D.

Department of Radiology, College of Medicine, The Catholic University of Korea,

222 Banpo-daero, Seocho-gu,

Seoul 06591, Korea.

Tel: (822) 2558-1443 • Fax: (822) 599-6771

E-mail: nyshin@catholic.ac.kr

Seung-Koo Lee M.D., Ph.D

Department of Radiology, Research Institute of Radiological Science, Yonsei University College of Medicine

50-1 Yonsei-ro, Seodaemun-gu,

Seoul 120-752, Korea.

Tel: (822) 2228-2373 • Fax: (822) 393-3035

E-mail: slee@yuhs.ac

**Supplementary materials**

Figure S1 Diagram of the striatal region-of-interest (ROI) that was used to quantitatively measure dopamine transporter (DAT) imaging.


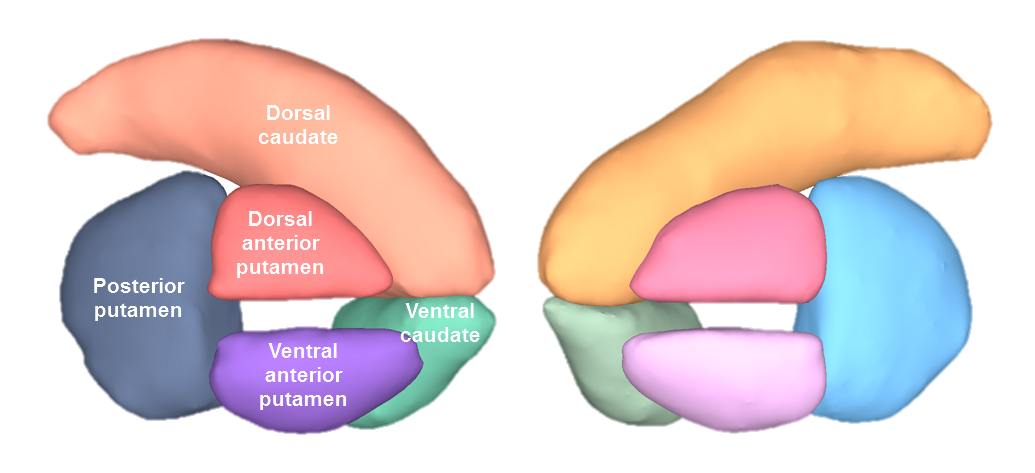


Table S1 Comparison of neuropsychological test results between the PD, DIP and control groups

| Cognitive Subdomains | | PD | DIP | Control | *P* value | *Post-hoc analysis* | |  |
| --- | --- | --- | --- | --- | --- | --- | --- | --- |
|  |  |  |  |  |  | ***P1*** | ***P2*** | ***P3*** |
| Attention | |  |  |  |  |  |  |  |
|  | Digit Span (forward) | 6.24 ± 1.40 | 5.52 ± 1.44 | 6.62 ± 1.32 | 0.003 | 0.045 | 0.476 | 0.002 |
|  | Digit Span (backward) | 3.58 ± 1.35 | 2.85 ± 1.39 | 3.84 ± 1.17 | 0.004 | 0.030 | 0.867 | 0.003 |
|  | Digit Span total | 9.82 ± 2.43 | 8.36 ± 2.57 | 10.27 ± 2.67 | 0.004 | 0.024 | 1.000 | 0.004 |
|  | Word Stroop test | 108.83 ± 10.67 | 103.76 ± 18.64 | 111.07 ± 4.27 | 0.032 | 0.155 | 0.977 | 0.028 |
|  | Color Stroop test | 72.64 ± 29.36 | 57.69 ± 29.57 | 83.88 ± 19.58 | <0.001 | 0.041 | 0.104 | <0.001 |
| Executive function | |  |  |  |  |  |  |  |
|  | Phonemic generative naming | 19.95 ± 10.27 | 15.21 ± 9.92 | 25.52 ± 11.47 | <0.001 | 0.154 | 0.025 | <0.001 |
|  | COWAT (animal) | 13.33 ± 3.35 | 11.82 ± 3.50 | 16.14 ± 4.40 | <0.001 | 0.177 | 0.001 | <0.001 |
|  | COWAT (supermarket) | 14.39 ± 5.23 | 13.00 ± 4.73 | 17.82 ± 5.59 | <0.001 | 0.641 | 0.003 | <0.001 |
| Verbal memory function | |  |  |  |  |  |  |  |
| SVLT |  |  |  |  |  |  |  |  |
|  | Free recall | 17.91 ± 4.69 | 15.94 ± 4.59 | 20.31 ± 3.88 | <0.001 | 0.116 | 0.017 | <0.001 |
|  | Delayed recall | 4.82 ± 2.64 | 3.94 ± 3.13 | 6.58 ± 1.82 | <0.001 | 0.321 | 0.001 | <0.001 |
|  | Recognition | 10.32 ± 1.67 | 9.64 ± 1.60 | 10.78 ± 1.13 | 0.005 | 0.106 | 0.350 | 0.004 |
| Visual memory function | |  |  |  |  |  |  |  |
|  | RCFT immediate recall | 12.08 ± 6.75 | 8.47 ± 6.21 | 15.17 ± 5.79 | <0.001 | 0.026 | 0.040 | <0.001 |
|  | RCFT delayed recall | 12.06 ± 6.34 | 8.26 ± 6.14 | 14.70 ± 5.15 | <0.001 | 0.010 | 0.071 | <0.001 |
|  | RCFT recognition | 8.86 ± 2.26 | 8.70 ± 2.21 | 9.67 ± 1.52 | 0.064 | NA | NA | NA |
| Visuospatial function | |  |  |  |  |  |  |  |
|  | RCFT copy | 30.33 ± 7.06 | 26.12 ± 9.19 | 33.96 ± 2.94 | <0.001 | 0.011 | 0.018 | <0.001 |
| Language and related function | |  |  |  |  |  |  |  |
|  | K-BNT | 41.94 ± 9.04 | 36.55 ± 11.94 | 46.51 ± 7.42 | <0.001 | 0.023 | 0.037 | <0.001 |

Values are expressed as means ± standard deviations. PD=Parkinson's disease; DIP=drug-induced parkinsonism; SVLT= Seoul Verbal Learning Test; COWAT= Controlled Oral Word Association Test; RCFT= Rey Complex Figure Test; K-BNT= Korean version of the Boston Naming Test.

^§^*P* values for comparison among the 3 groups. In the post-hoc analysis, *P*1 indicates p values for comparison between the PD and DIP groups, *P*2 for comparison between the PD and control groups and *P*3 for comparison between the DIP and control groups.

Table S2 Detailed information of regions demonstrating changed ALFF in the PD and DIP groups compared to the control group.

| Analysis | Contrast | Region | Talairach coordinates |  |  | Numbers of voxels | P value |
| --- | --- | --- | --- | --- | --- | --- | --- |
|  |  |  | x | y | z |  |  |
| ALFF | DIP > IPD | Right Cerebellum | 33 | -81 | -27 | 41 | 0.005 |
|  | IPD > Control | Left Middle Temporal Gyrus | -57 | -63 | 21 | 44 | 0.004 |
|  | IPD < Control | Right Insular Cortex | 45 | 6 | -9 | 77 | <0.001 |
|  |  | Right Cuneus | 6 | -72 | 21 | 292 | <0.001 |
|  |  | Left Lingual Gyrus | -21 | -75 | -12 | 51 | 0.002 |
|  | DIP > Control | Right Paracentral Lobule | -6 | -30 | 69 | 50 | 0.002 |
|  | DIP < Control | Right Insular Cortex | 45 | 6 | -9 | 55 | 0.001 |
|  |  | Right Middle Occipital Gyrus | 36 | -78 | 18 | 299 | <0.001 |

Table S3 Detailed information of regions demonstrating changed fALFF in the PD and DIP groups compared to the control group.

| Analysis | Contrast | Region | Talairach coordinates |  |  | Numbers of voxels | P value |
| --- | --- | --- | --- | --- | --- | --- | --- |
|  |  |  | x | y | z |  |  |
| fALFF | DIP > IPD | Right Caudate Nucleus | 18 | 24 | 12 | 86 | <0.001 |
|  |  | Left Caudate Nucleus | -18 | 18 | 9 | 50 | <0.001 |
|  | DIP < IPD | Left Middle Occipital Gyrus | -27 | -69 | 39 | 50 | <0.001 |
|  | IPD > Control | Right Inferior Temporal Gyrus | -54 | -21 | -24 | 67 | <0.001 |
|  |  | Right Middle Frontal Gyrus | 30 | 54 | 3 | 107 | <0.001 |
|  |  | Left Inferior Temporal Gyrus | -45 | -3 | -36 | 54 | <0.001 |
|  |  | Right Inferior Temporal Gyrus | 45 | 6 | -39 | 54 | <0.001 |
|  | IPD < Control | Left Rolandic Operculum | -63 | -21 | 15 | 78 | <0.001 |
|  |  | Right Cerebellum | 18 | -60 | -18 | 53 | <0.001 |
|  |  | Right Calcarine Sulcus | 12 | -84 | 15 | 91 | <0.001 |
|  |  | Right Middle Occipital Gyrus | 45 | -72 | 6 | 49 | <0.001 |
|  | DIP > Control | Right Putamen | 24 | 21 | -3 | 44 | 0.001 |
|  | DIP < Control | Right Precuneus | 18 | -63 | 24 | 65 | <0.001 |
|  |  | Left Middle Occipital Gyrus | 36 | -78 | 18 | 299 | 0.001 |

Table S4 Detailed information of regions demonstrating changed ReHo in the PD and DIP groups compared to the control group.

| Analysis | Contrast | Region | Talairach coordinates |  |  | Numbers of voxels | P value |
| --- | --- | --- | --- | --- | --- | --- | --- |
|  |  |  | x | y | z |  |  |
| ReHo | DIP > IPD | Right Olfactory Gyrus | 6 | 24 | -3 | 48 | 0.002 |
|  |  | Right Postcentral Gyrus | 45 | -33 | 60 | 52 | 0.002 |
|  | DIP < IPD | Left Precentral Gyrus | -48 | 3 | 45 | 85 | <0.001 |
|  |  | Left Middle Temporal Gyrus | -60 | -51 | 21 | 45 | 0.003 |
|  |  | Left Inferior Temporal Gyrus | -48 | 3 | -36 | 109 | <0.001 |
|  | IPD > Control | Right Middle Frontal Gyrus | 39 | 33 | 48 | 50 | 0.002 |
|  |  | Left Middle Temporal Gyrus | -60 | -51 | 21 | 60 | 0.001 |
|  |  | Right Cerebellum | 3 | -51 | -51 | 79 | <0.001 |
|  |  | Left Middle Temporal Gyrus | -57 | -48 | 0 | 60 | 0.001 |
|  |  | Right Angular Gyrus | 54 | -63 | 30 | 42 | 0.004 |
|  | IPD < Control | Right Middle Occipital Gyrus | 45 | -72 | 6 | 69 | <0.001 |
|  |  | Left Calcarine Sulcus | 0 | -75 | 15 | 362 | <0.001 |
|  |  | Right Postcentral Gyrus | 21 | -45 | 66 | 63 | 0.001 |
|  |  | Left Rolandic Operculum | -63 | -21 | 15 | 86 | <0.001 |
|  |  | Right Postcentral Gyrus | 60 | -6 | 21 | 42 | 0.004 |
|  | DIP > Control | Right Cerebellum | 3 | -54 | -51 | 74 | <0.001 |
|  | DIP < Control | Left Inferior Occipital Gyrus | -30 | -87 | -6 | 91 | <0.001 |
|  |  | Right Fusiform Gyrus | 27 | -57 | -15 | 56 | 0.001 |
|  |  | Right Cuneus | 18 | -63 | 21 | 59 | 0.001 |

Table S5 Result of DAT uptake in the PD and DIP groups

|  | Parkinson disease (N=60) | | Drug-induced parkinsonism (N=67) | | *p* value |
| --- | --- | --- | --- | --- | --- |
| Ventral caudate | 3.04±0.76 |  | 3.68±0.73 |  | <0.001 |
| Dorsal caudate | 2.12±0.62 |  | 2.53±0.65 |  | <0.001 |
| Posterior putamen | 2.33±0.59 |  | 4.51±0.71 |  | <0.001 |
| Anterior ventral putamen | 3.55±0.74 |  | 4.97±0.78 |  | <0.001 |
| Anterior dorsal putamen | 2.55±0.61 |  | 4.23±0.76 |  | <0.001 |

Values are expressed as means ± standard deviations.
